# Isolation of *C*. *difficile* Carriers Alone and as Part of a Bundle Approach for the Prevention of *Clostridium difficile* Infection (CDI): A Mathematical Model Based on Clinical Study Data

**DOI:** 10.1371/journal.pone.0156577

**Published:** 2016-06-03

**Authors:** Christos A. Grigoras, Fainareti N. Zervou, Ioannis M. Zacharioudakis, Constantinos I. Siettos, Eleftherios Mylonakis

**Affiliations:** 1 Infectious Diseases Division, Warren Alpert Medical School of Brown University, Providence, Rhode Island, United States of America; 2 School of Applied Mathematics and Physical Sciences, National Technical University of Athens, Athens, Greece; Cleveland Clinic, UNITED STATES

## Abstract

*Clostridium difficile* infection is the most common hospital-acquired infection. Besides infected patients, carriers have emerged as a key player in *C*. *difficile* epidemiology. In this study, we evaluated the impact of identifying and isolating carriers upon hospital admission on the incidence of CDI incidence and hospital-acquired *C*. *difficile* colonization, as a single policy and as part of bundle approaches. We simulated *C*. *difficile* transmission using a stochastic mathematical approach, considering the contribution of carriers based on published literature. In the baseline scenario, CDI incidence was 6.18/1,000 admissions (95% CI, 5.72–6.65), simulating reported estimates from U.S. hospital discharges. The acquisition rate of *C*. *difficile* carriage was 9.72/1,000 admissions (95% CI, 9.15–10.31). Screening and isolation of colonized patients on admission to the hospital decreased CDI incidence to 4.99/1,000 admissions (95% CI, 4.59–5.42; relative reduction (RR) = 19.1%) and led to 36.2% reduction in the rate of hospital-acquired colonization. Simulating an antimicrobial stewardship program reduced CDI rate to 2.35/1,000 admissions (95% CI, 2.07–2.65). In sensitivity analysis, CDI incidence was less than 2.32/1,000 admissions (RR = 62.4%) in 95% of 1,000 simulations. The combined bundle, focusing on reducing *C*. *difficile* transmission from colonized patients and the individual risk of these patients to develop CDI, decreased significantly the incidence of both CDI and hospital-acquired colonization. Implementation of this bundle to current practice is expected to have an important impact in containing CDI.

## Introduction

*Clostridium difficile* infection (CDI) is the most common hospital-acquired infection (HAI) in the US, currently constituting 12.1% of all HAIs[1]. The rising CDI incidence worldwide[1–3], along with the high morbidity and mortality of this infection[4, 5], emphasize the need for new preventive strategies[6]. Professional medical associations such as the Infectious Diseases Society of America, the American College of Gastroenterology, and the Society for Healthcare Epidemiology of America, recommend preventive policies that target CDI patients[7–9], including isolation of infected patients, and cleaning and disinfection of patient rooms and environmental surfaces. Moreover, the exposure to antibiotics or proton pump inhibitors(PPIs) poses high risk for developing CDI, both in hospital settings and the community[10–12].

A growing interest exists regarding the role of asymptomatically colonized patients [13, 14]. Indeed, recent studies suggest that at least 30% of patients with hospital-acquired CDI acquire the pathogen through direct or indirect contact with colonized patients [15]. According to Loo *et al*. the North American PFGE type 1 (NAP1) strain was identified to be responsible for almost 63% of the CDIs and 36% of the hospital acquired *C*. *difficile* colonization [16]. Also, patients colonized with toxinogenic *C*. *difficile* on hospital admission have an almost 6-fold higher risk to develop CDI compared to susceptible patients[17]. Our aim was first to model *C*. *difficile* transmission based on current published data regarding the mean CDI incidence and the contribution of *C*. *difficile* carriers. Thereafter, we used this model to examine the potential additive effectiveness of policies that target this specific patient population in decreasing both CDI and hospital-acquired colonization incidence.

## Methods

### Modeling the transmission dynamics of *C*. *difficile*

#### Transmission Dynamics

**Fig 1:**
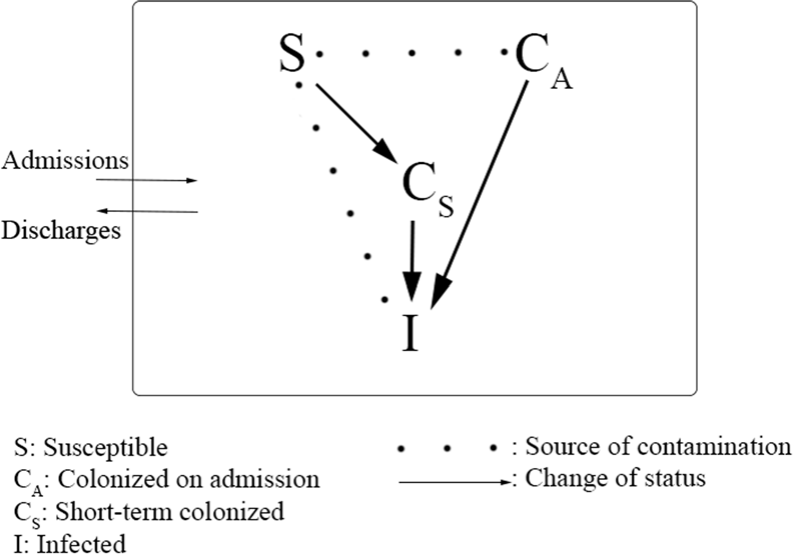
Schematic of the model describing the transmission dynamics of *C*. *difficile*.

The model describes the transmission dynamics of *C*. *difficile* in patients hospitalized in a 500-bed tertiary hospital (Fig 1). Upon hospital admission, patients were divided into 3 categories. The first consisted of infected patients (*I*) admitted with active CDI (≥3 loose stools/day in which toxinogenic *C*. *difficile* and/or its toxins were identified[8]). These patients were tested for CDI as per standard practice[8], and appropriate contact precautions were implemented for the duration of diarrhea[8].

The second category consisted of patients asymptomatically colonized with *C*. *difficile* (*C*_*A*_). These patients did not have diarrhea, but they harbored toxinogenic *C*. *difficile* and could progress to CDI during their hospital stay. The rate of progression was based on the percentage of infections that are expected to come from patients colonized on admission, assuming a uniform distribution of this rate throughout their hospital stay.

The third category consisted of the susceptible patients who were neither infected nor colonized on admission to the hospital (*S*). Those patients could develop CDI during their hospitalization after progressing through the intermediate stage of short-term colonization (*C*_*S*_). In order to become colonized, these patients had to acquire the pathogen through contact with patients with CDI or colonization, or through contact with the inanimate environment previously infested by colonized and/or infected patients. Those patients could develop CDI during their hospital stay with a rate that was modeled based on the reported risk and mean time of progression to CDI for this specific patient population. The aforementioned categories of patients sum up to the total number of patients *N*. (Eq 1)

$$N=S+C_{A}+C_{S}+I$$ Eq 1

The rate of admitted patients *i* was matched to that of discharges, deaths and transfers, *δ*_*i*_ and *δ* for infected and non-infected patients respectively, to keep the population in frequency dependent dynamics. (Eq 2)

$$iN=\delta (S+C_{A}+C_{S})+\delta_{i}I$$ Eq 2

The rate of fluctuation of the number of susceptible patients was determined estimating the difference in the number of new admissions of susceptible and the number of susceptible patients discharged, colonized by contact with infected, or colonized on admission patients. (Eq 3) Hereinafter, the set of coefficients *f* denote the effect of each intervention strategy, further explained in the section “Preventive Policies to Contain CDI”. The rate of contact between susceptible and infected patients and the likelihood of colonization as a result of their contact resided in the transmission coefficient ‘b_1_’ (Eq 3). Similarly, the transmission of the pathogen as a result of the contact between susceptible and colonized on admission patients was characterized by the coefficient ‘b_2_’ (Eq 3). According to the findings by Riggs *et al*., it can be deducted that *C*. *difficile* spores can be easily transmitted to health care workers by asymptomatic carriers, while at the same time, the environmental load of *C*. *difficile* was identical to ~60% of the concurrent stool samples [6]. Therefore, the combined probability to acquire *C*. *difficile* was integrated in the transmission coefficients ‘b_1_’ and ‘b_2_’ that were estimated so that the average simulated incidence rate approximates the observed incidence rate.

$$\frac{dS}{dt}=\varepsilon_{S}iN-\frac{\beta_{1}IS}{N}-\frac{f_{1}\beta_{2}C_{A}S}{N}-\delta S$$ Eq 3

The rate of change of the colonized on admission patients was the difference between the rate of colonized admissions and the rate of *C*_*A*_ patients discharged or developing symptoms of the infection. (Eq 4)

$$\frac{dC_{A}}{dt}=\varepsilon_{CA}iN-f_{2}\frac{r_{C_{A}I}}{t_{C_{A}I}}C_{A}-\delta C_{A}$$ Eq 4

Similarly, the rate of variation in the short-term colonized compartment was described by the resultant of the daily rates of change in the cardinality of *C*_*S*_ patients who were newly colonized, discharged or infected. (Eq 5)

$$\frac{dC_{S}}{dt}=\frac{\beta_{1}IS}{N}+\frac{f_{1}\beta_{2}C_{A}S}{N}-f_{2}\frac{r_{C_{S}I}}{t_{C_{S}I}}C_{S}-\delta C_{S}$$ Eq 5

The rate of change in the number of infected patients was calculated as the difference in the daily number of discharges and the new patients diagnosed with CDI.

$$\frac{dI}{dt}=\varepsilon_{I}iN+f_{2}\frac{r_{C_{A}I}}{t_{C_{A}I}}C_{A}+f_{2}\frac{r_{C_{S}I}}{t_{C_{S}I}}C_{S}-\delta_{I}I$$ Eq 6

#### Baseline parameterization

Baseline parameters are summarized in Table 1. Upon hospital admission, we considered that 0.3% of patients were diagnosed with CDI (*ε*_*I*_). This calculation was based on published administrative data from U.S. hospital discharges [18] regarding the percentage of patients who are admitted to the hospital with a primary diagnosis of CDI. Among the remaining patients, 10% were considered to be colonized with toxinogenic *C*. *difficile* based on the results of a recently published meta-analysis (*ε*_*CA*_) [17].

**Table 1:** Model Inputs. *C*. *difficile* = *Clostridium difficile*, CDI = *Clostridium difficile* infection, PCR = polymerase-chain reaction, R = reference.

Baseline Probabilities	Value (range)	Source
CDI Prevalence on Admission, U.S. (*ε*_*D*_)	0.003	[18]
CDI Incidence among U.S. Hospital Discharges	8.2 per 1,000 discharges	[20]
*C*. *difficile* Colonization Prevalence on Hospital Admission, North America	0.10 (0.071–0.134)	[17]
Mean Length of Stay for Non-Infected Patients (days) (1/d)	4.5	[25]
Mean Length of Stay Attributable to CDI (days) (1/d_i_)	2.9	[24, 39]
Risk of Short-Term Colonized Patients to Develop CDI During Hospital Stay (*r*_*SC*_)	0.60	[21]
Mean Time for Short-Term Colonized Patients to Develop CDI (days) (*t*_*SCD*_)	2	[22]
Percentage of CDIs coming from colonized on admission patients	0.341 (0.195–0.505)	[17]
**Probabilities assigned to Antimicrobial Stewardship Program**		
Relative Reduction of CDI Incidence after Implementation of an Antimicrobial Stewardship Program (*s*)	0.52 (0.38–0.62)	[36]
**Probabilities assigned to Screening and Contact Precautions**		
Sensitivity of PCR compared to toxinogenic culture (*c*_*1*_)	0.92 (0.91–0.94)	[33]
Compliance with contact precautions (*c*_*2*_)	0.772 (0.632–1.0)	[34]
Prevalence of contact precautions to admitted patients for MRSA or VRE colonization and/ or infection (*c*_*3*_)	0.047 (0.045–0.049)	[35]

An equal percentage of patients with hospital-acquired *C*. *difficile* colonization were considered to have acquired the pathogen through contact with colonized and infected patients under contact precautions, as shown by a study that used genotyping with multilocus variable number of tandem repeats to determine the transmission dynamics of *C*. *difficile* from asymptomatic carriers [15]. We considered that the risk of a susceptible patient to become colonized was independent from the receipt of antimicrobial agents based on the absence of difference that has been observed between the two groups[16, 19].

The CDI rate in our baseline model was based on the most recent estimate from U.S. hospital discharges, that is 8.2 per 1,000 hospital discharges[20]. Among incident infections, 34.1% was considered to come from patients colonized on admission who developed CDI during their hospital stay. This was based on a random effects meta-analysis that we did using data extracted for the purposes of our previous study regarding the prevalence of toxinogenic *C*. *difficile* colonization upon hospital admission and the risk for ensuing CDI in colonized and not colonized patients (S1 Fig)[17].

Patients with hospital-acquired *C*. *difficile* carriage were estimated to have a 60% risk to develop CDI during their remaining hospital stay as reported by Kyne *et al*.[21], with the mean time to infection being 2 days[22]. Because of the short time interval, and the absence of reliable clinical data, we assumed that short-term colonized patients could not transmit the pathogen in the short interim period between the establishment of colonization and the development of infection, a limitation of this study.

Finally, patients were considered to have an extended length of stay by 2.9 days due to CDI. This was based on the studies that compared the length of stay of CDI infected and non-infected patients taking into account the patients baseline risk of death and the time-varying effect of CDI[23, 24]. The length of stay for non-CDI patients was configured to resemble the mean US hospital stay of 4.5 days[25].

#### Mathematical model

The set of ordinary differential equations describing the aforementioned dynamics (Eqs 3–6) was transcribed into a stochastic model, in order to implement the Gillespie's direct method for epidemiological systems of finite size. This method has been employed by several studies modeling the transmission of *C*. *difficile* in hospital settings[26–29]. The simulations were performed using the “τ-leap method” proposed by Keeling *et al*[30]. The mean incidence was defined as the number of new infected patients per total population admitted. Because of lack of relevant clinical data, a uniform distribution was assumed for the daily rate of *C*. *difficile* transmission from colonized and infected patients during their entire hospital stay.

The values of those two parameters were retrospectively selected to produce the CDI incidence and the disease and short-term colonization source ratios as previously described.

### Preventive Policies to Contain CDI

#### I. Screening and Isolation of Colonized Patients

In addition to the current standard of care of the baseline model[8], admitted patients with no symptoms of CDI were considered as screened with real-time PCR for *C*. *difficile* toxins in either stool or rectal swabs (for those unable to provide stool specimens in a timely manner)[31]. Real-time PCR was used due to its increasing clinical use, high sensitivity and the need for a rapid turn-around time[32]. We considered a PCR sensitivity (*c*_*1*_) of 92%, an estimation derived from a meta-analysis regarding the sensitivity of PCR compared to the “gold standard” of toxinogenic culture[33]. Notably, the selection of the value of sensitivity is accordant to the value of CDI prevalence, as indicated by Deshpande *et al*.[32]. Patients detected as colonized were isolated using contact precautions for their entire hospitalization, due to existing uncertainty regarding the rate of spontaneous decolonization.

The mean rate of compliance (*c*_*2*_) with the use of gowns and gloves in patients under contact precautions is calculated to be 77.2% [34], providing a reasonable rate of success for this measure. Of note, 4.7% of patients were considered to be already under contact precautions for reasons other than *C*. *difficile* (MRSA and/or VRE colonization or infection)[35], and thus the risk reduction due to screening and isolation for *C*. *difficile* was not applicable to them (*c*_*3*_). This risk reduction was also not applicable to 8% of patients that, although colonized with toxinogenic *C*. *difficile* strains, were not detected by PCR (1-*c*_*1*_). Of note, we assumed that patients colonized on admission were isolated in a timely manner with no breakthrough transmission. Also, we considered MRSA/VRE isolation effective for the control of transmission from *C*. *difficile* carriers. The quantitative effect of the screening and isolation of colonized patients (*f*_*1*_) was expressed by Eq 7

$$f_{1}=(1-c_{1})+c_{3}c_{1}+c_{1}(1-c_{2})(1-c_{3})$$ Eq 7

#### II. Screening and Isolation of Colonized Patients as Part of Bundle Approaches

The policy of screening and isolation of colonized patients was further combined, as part of different bundle approaches, with strategies that had the potential to decrease the risk of CDI in colonized patients. First, it was combined with the implementation of an antimicrobial stewardship program, restrictive or persuasive. Although antimicrobial stewardship programs have extensive differences, in our model this strategy was considered to be able to lead to a 52% overall reduction in CDI rate (*s*), based on the results of a recent meta-analysis published by Feazel *et al*. that analyzed data from 16 studies[36]. The effect of an antimicrobial stewardship program to CDI prevention was considered to be fully sustained during the simulated time period, based on the findings by Cook *et al*., reporting a decrease of the nosocomial CDI rates by 42.6% (P = 0.005) between 2003 and 2010 that was associated with a decrease in total antimicrobial use by 62.8% (P<0.0001) [37]. We assumed that the individual reduction in CDI risk was equal among patients colonized on admission and those newly colonized. (Eq 8)

$$f_{2}=(1-s)$$ Eq 8

### Statistical Analysis

A probabilistic sensitivity analysis on the effectiveness of all combined strategies was performed to validate the robustness of the results. We performed 1,000 simulations with random selection of the value of each parameter from the reported confidence intervals (Table 1). For the compliance with contact precautions, we used as lower limit the reported mean compliance with the use of gowns, gloves together with hand washing before and after contact with isolated patients and as upper limit the ideal 100% compliance. To examine the effectiveness of the applied interventions with regard to the different virulence of less epidemic *C*. *difficile* strains, we also varied the risk of SC patients to develop into CDI, with the lower limit set to half the *r*_*SC*_, based on the methodology utilized by Starr *et al*.[38]. Similarly, the coefficient of PCR sensitivity (c_1_) was set to half, to take into consideration the potential reduced sensitivity of PCR test to detect asymptomatic colonized patients [38]. All the parameters were assumed to follow a uniform distribution to achieve more conservative estimations. The MATLAB and Statistics Toolbox Release 2015b (The MathWorks Inc., Natick, MA) was used for the entire analysis.

## Results

Our model simulated the transmission dynamics of CDI in a 500-bed hospital. This transmission dynamics model was run for 1,000 days during which 110,480 admissions occurred. Based on the estimates detailed under *Methods*, the mean admission rate of colonized patients was 10.99 patients per day and that of infected patients was 0.35 per day.

Based on the input values of the baseline parameters and the disease source ratio for the patients who were not colonized at the time of hospital admission, we estimated a transmission coefficient of 0.1059 (b_1_ = 0.1059) that characterized the contacts of susceptible patients with infected patients under contact precautions. The relevant transmission coefficient for the contacts of susceptible on admission patients with colonized on admission patients who were not under contact precautions was estimated to be 0.007 (b_2_ = 0.007). Indeed, the CDI incidence in this baseline scenario was 6.18 per 1,000 admissions (95% CI, 5.72–6.65), simulating the reported rate of CDI in 2010 as estimated from data on U.S. hospital discharges[20].

Screening of patients at the time of hospital admission with PCR and isolation of those colonized, as a single additive policy to the standard practice, reduced CDI incidence to 4.99 per 1,000 admissions (95% CI, 4.59–5.42; RR = 19.1%). Applying this policy as part of a bundle approach combined with an antimicrobial stewardship program had effectiveness in reducing CDI incidence. Specifically, CDI incidence reduced to 2.35 per 1,000 admissions (95% CI, 2.07–2.65; RR = 61.88%) with the addition of an antimicrobial stewardship program.

Given the importance of the health-care system for the acquisition of *C*. *difficile*, we also estimated the effectiveness of the above policies in reducing the number of patients with hospital-acquired colonization. In the baseline model, the rate of hospital-acquired colonization was estimated to be 9.72 per 1,000 admissions (95% CI, 9.15–10.31). The implementation of contact precautions for patients found to be colonized on admission reduced the relevant rate to 6.20 per 1,000 admissions (95% CI, 5.75–8.5; RR = 36.22%). As expected, the bundle that combined all studied policies was the most effective policy and reduced the number of newly colonized patients to 4.22 every 1,000 admissions (95% CI, 3.85–4.61; RR = 56.58%). The aforementioned results are presented in Table 2.

**Table 2:** Summary estimates of CDI incidence per 1,000 admissions and rate of newly colonized with *C*. *difficile* patients per 1,000 admissions. *C*. *difficile* = *Clostridium difficile*, CDI = *Clostridium difficile* infection, CIs = confidence intervals, R = reference.

	**CDI Incidence Rate per 1,000 admissions (95% CIs)**	**Relative Reduction (%)**
Baseline	6.18 (5.72–6.65)	-
Screening and Contact Precautions	4.99 (4.59–5.42)	19.1
Screening, Contact Precautions and Antimicrobial Stewardship	2.35 (2.08–2.65)	61.88
	**Newly colonized patients per 1,000 admissions (95% CIs)**	**Relative Reduction (%)**
Baseline	9.72 (9.15–10.31)	-
Screening and Contact Precautions	6.21 (5.75–8.5)	36.22
Screening, Contact Precautions and Antimicrobial Stewardship	4.22 (3.85–4.61)	56.6

**Fig 2:**
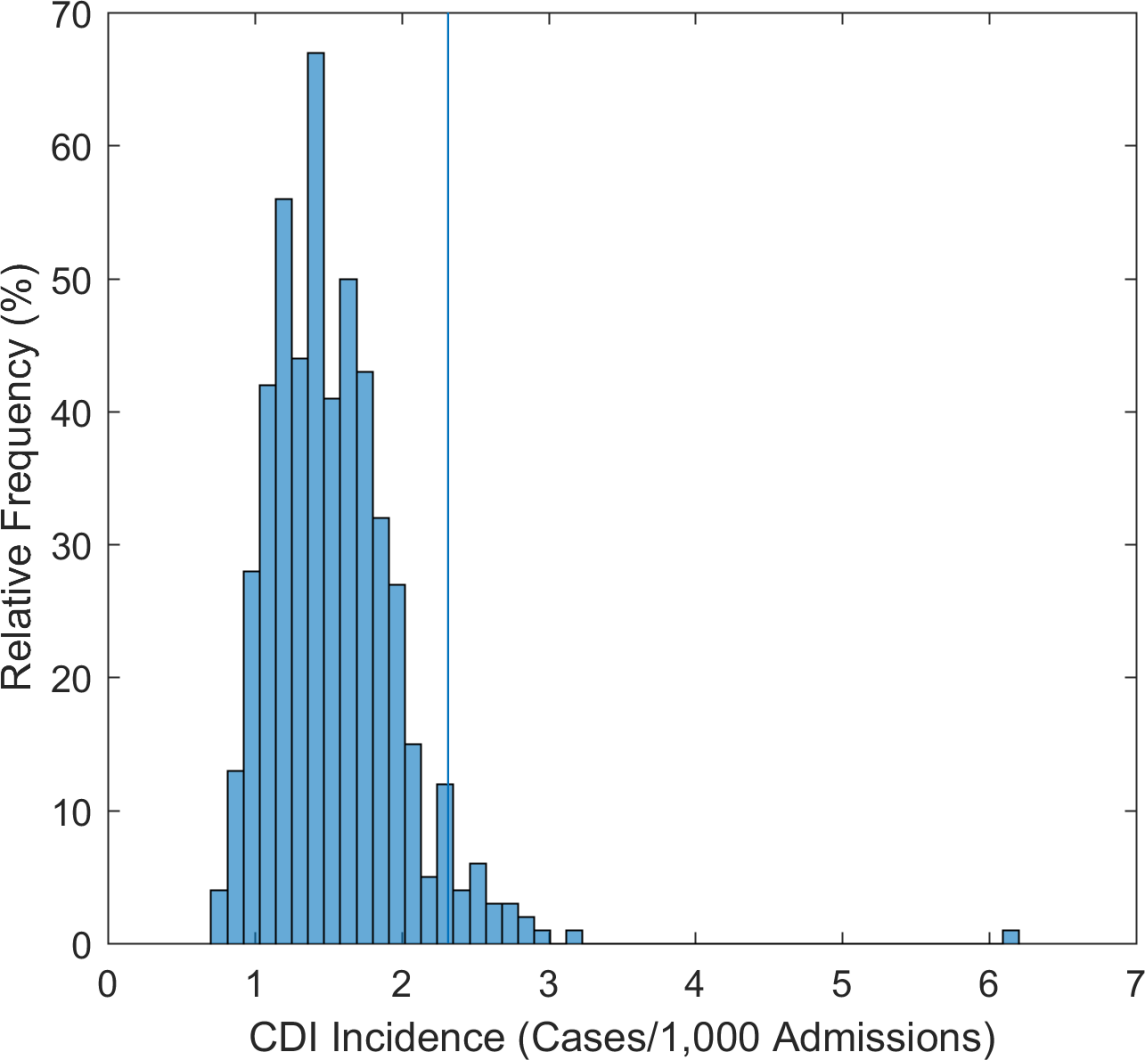
Probabilistic sensitivity analysis. Histogram of the incidence rate in 1,000 simulations. In 95% of simulations the incidence rate is below 2.32 per 1,000 admissions (dashed line), corresponding to a 62.4% reduction of the baseline CDI rate.

Finally, our model was run 1,000 times to validate the robustness of the estimated effectiveness of the combined bundle in decreasing CDI incidence, with random value selection of the effectiveness of each individual policy within the pre-specified ranges mentioned in the *Methods*. This probabilistic sensitivity analysis showed that in 95% of 1,000 simulations the combination of all policies managed to reduce the baseline incidence to lower than 2.32 per 1,000 admissions, equivalent to a 62.4% reduction compared to the baseline, confirming the robustness of the result reported in our base case analysis (Fig 2).

## Discussion

In this study, we examined the effectiveness of preventive policies targeting asymptomatically colonized patients in containing CDI. To increase the applicability of our estimations, we first constructed a model that simulated the CDI incidence as reported among U.S hospital discharges[20], and we fitted the model parameters using data reported by clinical studies. The identification and isolation of patients colonized with *C*. *difficile* on hospital admission had a significant effect in reducing CDI rate. Implementing this policy as part of a bundle approach with an antimicrobial stewardship program further reduced the rate of infection and of newly colonized patients by 61.88% and 56.58%, respectively. Our estimation regarding the effectiveness of this bundle approach was robust under sensitivity analysis, which indicated that in 95% of simulations CDI rate was 62.4% lower than the baseline rate.

Several published studies presented different approaches contributing significantly to a better knowledge of *C*. *difficile* epidemiology in a hospital setting. Starr *et al*. based their model on the assumption that, under normal circumstances, individuals are immune from colonization by *C*. *difficile* [38], something that does not reflect our current knowledge on the epidemiology of *C*. *difficile*, since it was demonstrated by Loo *et al*. that colonization is not associated with use of antimicrobial agents [40]. The studies by Yakob *et al*. [28] and Lanzas *et al*. [27] failed to display a decreased CDI incidence rate as a result of the cessation of antimicrobial treatment, significantly diverging from the recorded benefits of antimicrobial stewardship programs [36]. Our study provides realistic estimates of the combined interventions of isolating the colonized patients on admission and the enforcement of an antimicrobial stewardship program.

Asymptomatic carriers have been tracked as the source of almost 30% of CDIs in hospitals with infection control programs targeting only infected patients [15]. Indeed, asymptomatic carriers have been found to have a significant rate of skin and environmental contamination [6, 41]. However, there are no policies for patients colonized with *C*. *difficile*, and applying contact precaution measures in this patient population is a field under examination [42, 43]. In this context, our study provides the first comprehensive evidence that screening for asymptomatic carriage and enforcing contact precautions in an acute care hospital in the US should be considered. Indeed, our study found that isolation of *C*. *difficile* carriers on hospital admission managed to reduce CDI incidence by 29.2% compared to the baseline scenario where only patients with CDI were isolated. Furthermore, we applied contact precautions as part of bundle approaches that also took into account current evidence regarding the risk of patients colonized with *C*. *difficile* upon hospital admission to develop CDI during their hospitalization[17]. These policies have not been considered in previous modeling approaches[26], as colonized patients were considered protected from subsequent infection[44], and therefore these policies lacked the theoretical justification to be examined.

Our model integrates also the implementation of an antimicrobial stewardship program, which is currently the preventive policy for which the highest level of evidence exists regarding its effectiveness in reducing CDI rate[36]. Even though, the effect of antimicrobial treatment has been examined in other detailed models on CDI[27–29] and impact of cessation of antimicrobial treatment was included in some of those models the impact of an antimicrobial stewardship program was not verified in those studies. The application of an antimicrobial stewardship program managed in combination with other policies to reduce the rate of CDI by 61.9%, which is significantly higher from the reported 52% when it is the only additive policy.

Interestingly, even when all strategies were combined, a zero incidence rate was not achieved. This should be expected because no intervention is 100% effective and lapses in the implementation of interventions should be considered. Also, even when all policies were implemented, infected and colonized patients continued to be admitted to the hospital from the community at a stable rate. However, the significant decrease in the rate of hospital-acquired colonization that was observed in our model after the implementation of the combined bundle approach is particularly promising for a further long-term decrease of CDI rate, through a decrease of the rate of admission of both infected and colonized patients. Moreover, effective control of *C*. *difficile* epidemiology in other potential sources of *C*. *difficile*, such as the long-term care facilities[45], is needed.

Of note, although we evaluated a number of potential preventive strategies, we did not include the administration of probiotics[46], and fecal transplantation[47]. The use of probiotics was not considered due to their ambivalent results, with the most current evidence questioning their efficacy in CDI prevention[48]. On the other hand, the administration of fecal microbiota, although remarkably effective in prevention of recurrent episodes, has not been studied yet as a strategy to prevent initial CDI episodes. To alter the risk of colonized patients to progress to infection, radical approaches need to be considered. Such an approach would be the prophylactic administration of metronidazole to colonized patients, based on its recently shown effectiveness to reduce the risk of CDI in patients who are on antibiotics[45]. However, data on metronidazole were based on a retrospective study and have not been confirmed in randomized trials, and thus clinical trials are needed before the application of this strategy becomes justified.

It should be noted that our study could underestimate the results of isolation methods, since it assumes that contact precautions do not alter the risk of colonized on admission patients to develop CDI, since, at least some of those patients, will develop CDI from a different strain. Also, as all models, our analysis is based on assumptions that are clearly stated in the *Methods*. Even though, the selected model was heavily parameterized, it depicted in a realistic manner, the transmission network of the pathogen and the accuracy of the preventive policies. Importantly, the parameter values and their confidence intervals were either extracted from clinical studies, or estimated to fit the average reported CDI prevalence. To avoid bias in favor of our study, we considered a constant rate of admission of colonized and infected, despite the expected decrease due to the beneficial effect of the interventions.

In summary, the simulated bundle approach directed towards reducing the risk of colonized patients to develop CDI and transmit *C*. *difficile* had a significant effect in reducing CDI rate. The lack of trials examining the effectiveness of preventive bundles targeted towards asymptomatic *C*. *difficile* carriers makes the modeling evaluation of their potential to be of particular importance. However, clinical trials are the necessary next step to confirm those estimations, while the cost-benefit remains to be studied.

## Supporting Information

S1 File Forest Plot of Prevalence of *C*. *difficile* Infections Originating from Patients Colonized with *C*. *difficile* on Hospital Admission. (PDF)

S1 Fig Proportional Distribution of Patients over Time. (TIF)
