# Supplementary material for: Isolation of C. difficile Carriers Alone and as Part of a Bundle Approach for the Prevention of Clostridium difficile Infection (CDI): A Mathematical Model Based on Clinical Study Data
Source: PLoS One. 2016 Jun 3;11(6):e0156577. doi: 10.1371/journal.pone.0156577 (PMC4892551; doi:10.1371/journal.pone.0156577)
Supplement: S1 File — (PDF) [file pone.0156577.s001.pdf]

Supplementary Appendix

Supplement to: Isolation of C. difficile carriers alone and as part of a bundle approach for the prevention of Clostridium difficile Infection (CDI): A Mathematical Model Based on Clinical Study Data

Ioannis M. Zacharioudakis, M.D., Fainareti N. Zervou, M.D., Christos A. Grigoras, M.Sc., Constantinos I. Siettos, Ph.D., Eleftherios Mylonakis, M.D, Ph.D.

Table of Contents

Figure S1………………………………………………………………… 3

References……………………………………………………………...4

Figure S1. Forest Plot of Prevalence of *C. difficile* Infections Originating from Patients Colonized with *C. difficile* on Hospital Admission.

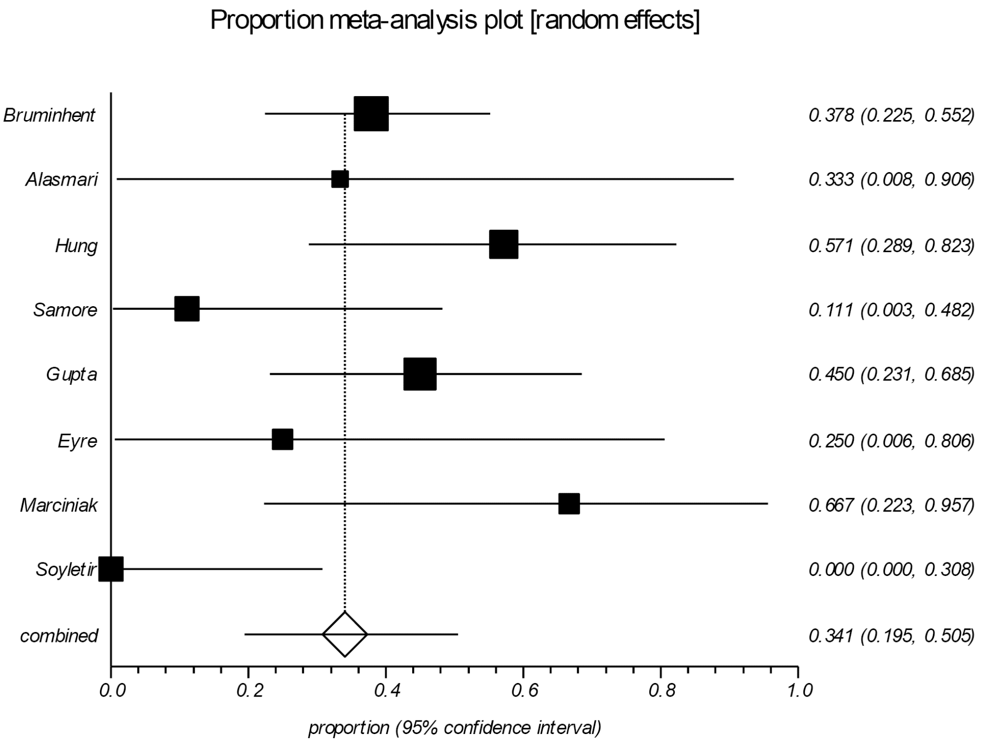

References of Studies Included in the Random-Effects Meta-Analysis of Prevalence of *C. difficile* Infections Originating from Patients Colonized with *C. difficile* on Hospital Admission.

1. Bruminhent J, Wang ZX, Hu C, et al. Clostridium difficile colonization and disease in patients undergoing hematopoietic stem cell transplantation. Biol Blood Marrow Transplant **2014**; 20(9): 1329-1334.

2. Alasmari F, Seiler SM, Hink T, Burnham CA, Dubberke ER. Prevalence and risk factors for asymptomatic Clostridium difficile carriage. Clin Infect Dis **2014**; 59(2): 216-222.

3. Hung YP, Tsai PJ, Hung KH, et al. Impact of toxigenic Clostridium difficile colonization and infection among hospitalized adults at a district hospital in southern Taiwan. PLoS One **2012**; 7(8): e42415.

4. Samore MH, DeGirolami PC, Tlucko A, Lichtenberg DA, Melvin ZA, Karchmer AW. Clostridium difficile colonization and diarrhea at a tertiary care hospital. Clin Infect Dis **1994**; 18(2): 181-187.

5. Gupta S, Mehta V, Herring T, et al. A Large Prospective North American Epidemiologic Study of Hospital-Associated Clostridium difficile Colonization & Infection. In: International Clostridium difficile Symposium, Bled, Slovenia, 22 September 2012. Abstract O20.

6. Eyre DW, Griffiths D, Vaughan A, et al. Asymptomatic Clostridium difficile colonisation and onward transmission. PLoS One **2013**; 8(11): e78445.

7. Marciniak C, Chen D, Stein AC, Semik PE. Prevalence of Clostridium difficile colonization at admission to rehabilitation. Arch Phys Med Rehabil **2006**; 87(8): 1086-1090.

8. Soyletir G, Eskiturk A, Kilic G, Korten V, Tozun N. Clostridium difficile acquisition rate and its role in nosocomial diarrhoea at a university hospital in Turkey. Eur J Epidemiol **1996**; 12(4): 391-394.
