# Supplementary figures and images for: Isolation of C. difficile Carriers Alone and as Part of a Bundle Approach for the Prevention of Clostridium difficile Infection (CDI): A Mathematical Model Based on Clinical Study Data
Source: PLoS One. 2016 Jun 3;11(6):e0156577. doi: 10.1371/journal.pone.0156577 (PMC4892551; doi:10.1371/journal.pone.0156577)

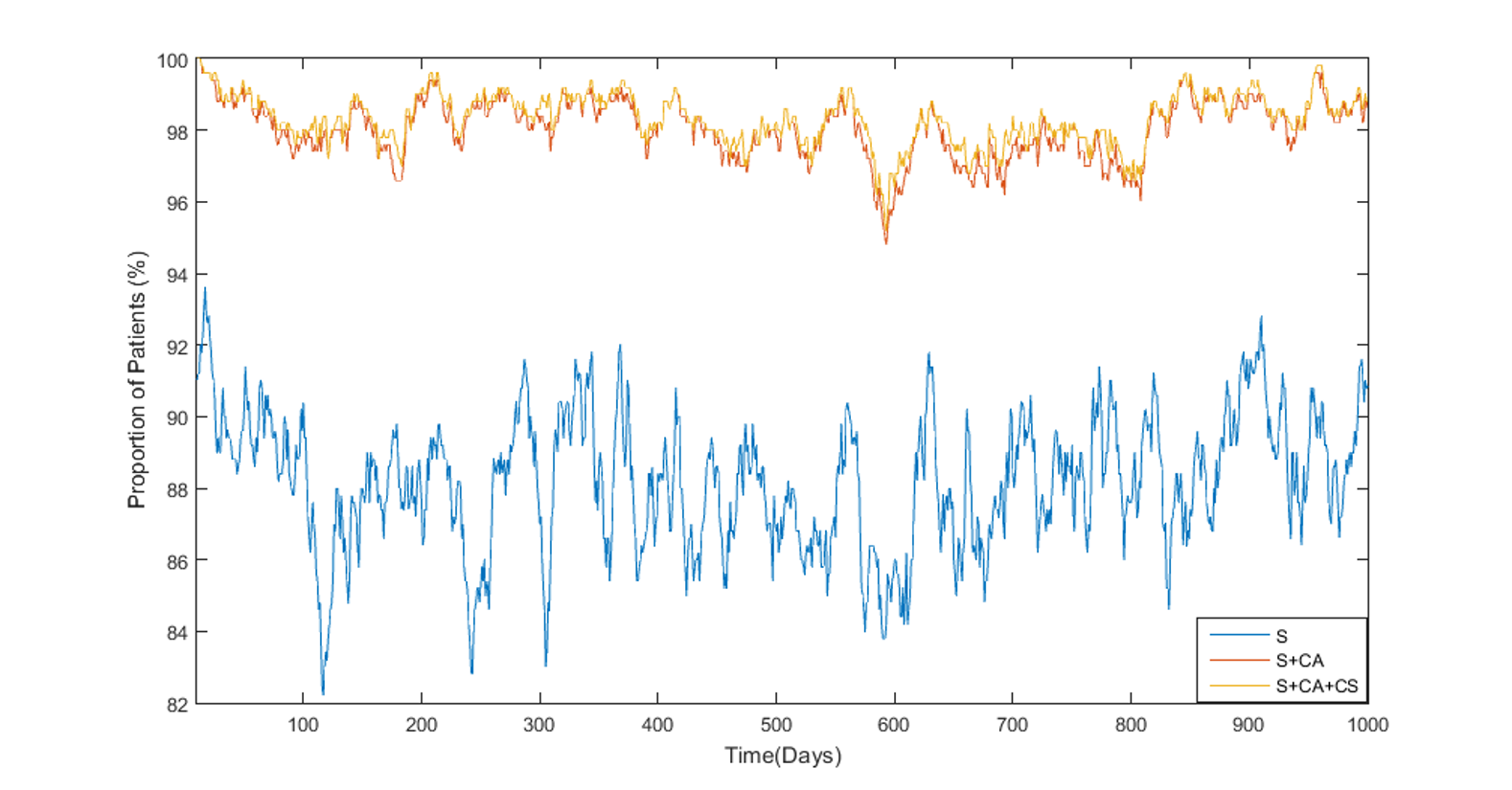

Supplement: S1 Fig — (TIF) [file pone.0156577.s002.tif]
